# Oxidative Stress-Induced Axon Fragmentation Is a Consequence of Reduced Axonal Transport in Hereditary Spastic Paraplegia *SPAST* Patient Neurons

**DOI:** 10.3389/fnins.2020.00401

**Published:** 2020-05-07

**Authors:** Gautam Wali, Erandhi Liyanage, Nicholas F. Blair, Ratneswary Sutharsan, Jin-Sung Park, Alan Mackay-Sim, Carolyn M. Sue

**Affiliations:** ^1^Department of Neurogenetics, Kolling Institute of Medical Research, The University of Sydney, Sydney, NSW, Australia; ^2^Sydney Medical School, The University of Sydney, Sydney, NSW, Australia; ^3^Griffith Institute for Drug Discovery, Griffith University, Nathan, QLD, Australia; ^4^Queensland Brain Institute, The University of Queensland, Brisbane, QLD, Australia; ^5^Department of Experimental Animal Research, Biomedical Research Institute, Seoul National University Hospital, Seoul, South Korea

**Keywords:** hereditary spastic paraplegia, *SPAST*, axon transport, peroxisomes, axon degeneration, epothilone D, noscapine

## Abstract

Hereditary spastic paraplegia (HSP) is a group of inherited disorders characterized by progressive spasticity and paralysis of the lower limbs. Autosomal dominant mutations in *SPAST* gene account for ∼40% of adult-onset patients. We have previously shown that *SPAST* patient cells have reduced organelle transport and are therefore more sensitive to oxidative stress. To test whether these effects are present in neuronal cells, we first generated 11 induced pluripotent stem (iPS) cell lines from fibroblasts of three healthy controls and three HSP patients with different *SPAST* mutations. These cells were differentiated into FOXG1-positive forebrain neurons and then evaluated for multiple aspects of axonal transport and fragmentation. Patient neurons exhibited reduced levels of *SPAST* encoded spastin, as well as a range of axonal deficits, including reduced levels of stabilized microtubules, lower peroxisome transport speed as a consequence of reduced microtubule-dependent transport, reduced number of peroxisomes, and higher density of axon swellings. Patient axons fragmented significantly more than controls following hydrogen peroxide exposure, suggesting for the first time that the *SPAST* patient axons are more sensitive than controls to the deleterious effects of oxidative stress. Treatment of patient neurons with tubulin-binding drugs epothilone D and noscapine rescued axon peroxisome transport and protected them against axon fragmentation induced by oxidative stress, showing that *SPAST* patient axons are vulnerable to oxidative stress-induced degeneration as a consequence of reduced axonal transport.

## Introduction

Hereditary spastic paraplegia (HSP) is an inherited neurological disorder that predominantly affects the corticospinal tracts, leading to lower limb weakness, spasticity, and paralysis (Salinas et al., 2008). Mutations in the *SPAST* gene are the most common cause of HSP, accounting for over 40% of autosomal dominant cases (Vandebona et al., 2012; Ruano et al., 2014). Spastin, the protein encoded by *SPAST*, is a microtubule severing protein that helps control microtubule stability and the cellular processes dependent on these structures, including organelle transport (Errico et al., 2002; McDermott et al., 2003). Histopathological studies suggest that deficits in organelle transport might be responsible for degeneration of the corticospinal tract axons, a common pathological feature of *SPAST* HSP cases (McDermott et al., 2000; Wharton et al., 2003; Orlacchio et al., 2004; Salinas et al., 2008; Duning et al., 2010), although this hypothesis has not been tested.

Multiple studies have consistently indicated that neurons from *SPAST* patients, post mortem and *in vitro*, have deficits in axon transport. For example, post-mortem spinal cord analyses of two patients with missense and frameshift *SPAST* mutations showed distinct axonal swellings containing abnormal neurofilament and accumulated mitochondria (Kasher et al., 2009). In three *SPAST* HSP cases, mitochondria were distributed more densely around the periphery of the neuronal soma of lower and upper motor neurons, in contrast to being uniformly distributed throughout the neuronal soma in healthy controls (McDermott et al., 2003). Similarly, induced pluripotent stem (iPS) cell-induced neurons from a patient with a mutation in intron 4 of the *SPAST* gene (c.683-1G > T) had abnormal axon swellings containing mitochondria and stable microtubules (i.e., acetylated α-tubulin) (Denton et al., 2014). Patient axons displayed reduced mitochondria motility, with fewer moving in retrograde compared to control axons (Denton et al., 2014). In a second study, glutamatergic neurons differentiated from iPS cells from two patients with the same non-sense mutation in the *SPAST* gene (c.1684C > T) (p.R562X), also had fewer axon mitochondria moving in retrograde compared to controls (Havlicek et al., 2014). Restoring axonal transport in *SPAST* HSP patients is therefore a promising therapeutic target.

The unique architecture of neurons, with axons extending up to 1 m in length, makes them more dependent on microtubules for organelle transport than other cell types. Unlike smaller cells and neuronal cell bodies that depend on both actin and microtubule cytoskeletons for organelle transport, organelle transport within axons is primarily dependent on microtubules (Barlan and Gelfand, 2017). Therefore, it would be expected that any condition that reduces microtubules would have a larger impact on neurons than other cell types. We have previously shown that olfactory neurosphere (ONS)-derived cells from *SPAST* patients have reduced levels of stabilized microtubules, leading to reduced organelle transport and thereby making them more sensitive to oxidative stress (Wali et al., 2016). In the present study, to test if these deficits are present in neurons and measure the impact the deficits have on neuronal function, we first generated six iPS cell lines from three *SPAST* patients and five iPS cell lines from three healthy controls. These were differentiated into forebrain neurons to act as a model for HSP and will here on be referred to as patient and control neurons. We then characterized several aspects of the axon transport and showed that patient neurons had less stabilized microtubules, reduced microtubule-dependent peroxisome transport, reduced number of peroxisomes, and a higher density of axon swellings. We found that patient axons exhibited increased axon fragmentation when compared to healthy control neurons. Finally, we show that two tubulin-binding drugs, epothilone D and noscapine, can rescue microtubule-dependent peroxisome axon transport in patient axons and protect them against the effects of oxidative stress. This represents a significant step toward the development of effective treatments for *SPAST-*related HSP.

## Materials and Methods

Detailed methods are in Supplementary Material.

### Participants

Hereditary spastic paraplegia patients in this study were reviewed and examined by a movement disorders specialist (CS). All patients exhibited similar clinical symptoms, typical of adult onset HSP, and were subsequently identified with *SPAST* mutations. Details of their clinical phenotypes are reported elsewhere (Vandebona et al., 2012) and the mutation identified in each patient is listed in Supplementary Table S1. Our study involving human participants was reviewed and approved by Human Research Ethics Committee affiliated to the Northern Sydney Local Health District, New South Wales government, Australia. The ethics committee reference number: RESP/15/314. The patients/participants provided their written informed consent to participate in this study.

### Generation and Characterization of Induced Pluripotent Stem Cell Lines

Skin fibroblasts were cultured and reprogrammed to iPS cells using the Sendai virus approach. iPS cell pluripotency was assessed by quantifying the expression of SSEA-4 and Sox2 by flow cytometry and immunostaining. TaqMan^®^ hPSC Scorecard (Thermofisher, A15872) assay was used to predict tri-lineage differentiation potential. Differentiation ability of iPS cells to the three germ layers was assessed by spontaneous differentiation of iPS cells to embryoid bodies (Abraham et al., 2010) and the expression of β III-tubulin, GATA4, and brachyury.

### Neuronal Differentiation of Induced Pluripotent Stem Cell Lines

All iPS cell lines were differentiated to FOXG1-positive forebrain neurons following a previously published protocol (Chambers et al., 2009; Maroof et al., 2013). Confirmation of neuronal differentiation was quantified in the cultures by immunofluorescence labeling with neuronal marker: β III-tubulin; synaptic marker, SYN1; and forebrain neuron marker, FOXG1.

### Phenotyping of Differentiated Patient Derived Neurons

(A) Spastin protein expression was assessed in all patient and control differentiated neuronal cultures using western blot analysis. (B) Axon swellings were quantified in images of axons immune-labeled with β III-tubulin. (C) Peroxisome imaging and analysis in living cells were quantified in patient and control cell lines using automated imaging and analysis as described previously (Wali et al., 2016). To test the effect of tubulin-binding drugs on peroxisome trafficking speeds, we quantified peroxisome speed in untreated control axons, untreated patient axons, and in patient axons treated with the tubulin-binding drugs, 2 nM epothilone D or 10 μM noscapine for 24 h prior to live cell imaging (Fan et al., 2014). (D) The effect of oxidative stress on axon fragmentation was quantified in axons exposed to H_2_O_2_. To induce oxidative stress, we used 20 μM H_2_O_2_, that causes axon fragmentation but not cell death (Bilsland et al., 2010; Fang et al., 2012). Patient and control axons were treated with H_2_O_2_ for 3, 6, and 12 h with media change every hour, after which axon degeneration was quantified by image analysis by measuring degeneration index (DI) as described previously (Sasaki et al., 2009). To determine whether the tubulin-binding drugs could rescue axon degeneration induced by oxidative stress, patient cells were treated with 2 nM epothilone D or 10 μM noscapine for 24 h prior to H_2_O_2_ treatment and DI was measured. Axon degeneration was measured as described previously (Sasaki et al., 2009). Briefly, bright-field images were binarized so that pixel intensity of the axon regions was black and all other regions white. The total number of black pixels was defined as the “total axon area.” The degenerated axons were detected using the ImageJ particle analyzer module and quantified the area of small fragments (size = 20–10,000 pixels). DI was calculated as the ratio of fragmented axon area over total axon area. This assay generates a score of 0 for completely intact axons and 1 for completely fragmented axons.

### Statistical Analysis

Data are expressed as mean ± SEM. SPSS Statistics Version 24 (IBM), Prism (GraphPad), and Stata IC Version 12.1 software (STATA Corp., College Station, TX, United States) were used to analyze data and plot graphs. In all graphs, the individual clones from the same cell lines have the same shape and are shown as filled or patterned.

## Results

### Generation of iPS Cell Lines From Patient and Control-Derived Skin Fibroblasts

**FIGURE 1:**
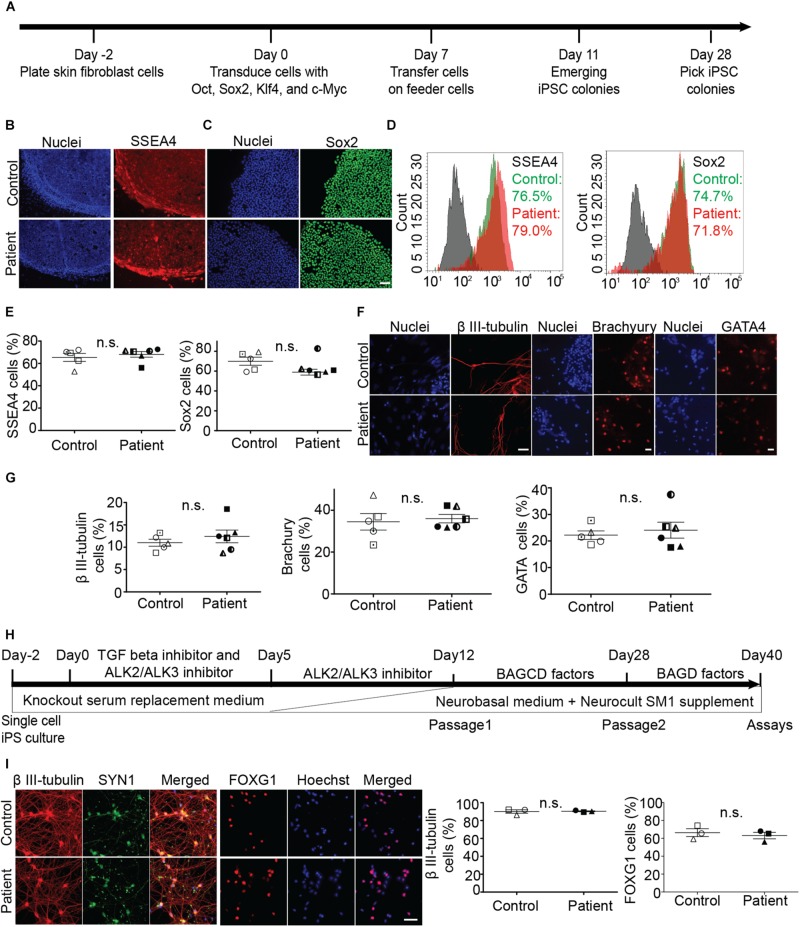
Generation and characterization of iPS cell lines and differentiation into neurons. **(A)** Timeline of reprogramming skin fibroblasts to iPS cells. **(B–E)** Expression of pluripotency markers SSEA-4 and Sox2 in patient and control iPS cells and group means (+/− SEM). **(D)** Flowcytometry based analysis of control and patient cells. **(F,G)** Embryoid body cells expressing three germ layer markers: β III-tubulin (ectoderm), brachyury (mesoderm), GATA4 (endoderm). **(H)** Timeline of iPS cell differentiation to forebrain neurons. Brain-derived neurotrophic factor (B), ascorbic acid (A), glial cell-derived neurotrophic factor (G), DAPT (D), and dibutyryl cAMP (C). **(I)** Expression of neuronal differentiation markers and quantification of neurons immuno-labeled for β III-tubulin and FOXG1 expression. Scale bar: 50 microns.

Fibroblasts were reprogrammed to iPS cells using the Sendai virus system (Figure 1A). We established five control and six patient iPS cell lines (multiple clones, details in Supplementary Table S1). All iPS cell lines expressed pluripotency markers SSEA4 and SOX2 (Figures 1B–E). Scorecard gene expression assay demonstrated that all iPS cell lines had gene expression associated with pluripotency and predicted differentiation capacity into neuroectoderm lineage (Supplementary Figure S1). Differentiation ability of iPS lines was confirmed by inducing embryoid body differentiation. iPS cell lines differentiated into cells positive for all germ layer markers, β III-tubulin, brachyury, and GATA-binding protein 4 (Figures 1F,G). No difference was seen in the pluripotency or differentiation ability between patient and control iPS cell lines (Figures 1E,G). No iPS cell lines had any chromosomal abnormality (Supplementary Figure S2A). The presence of *SPAST* mutations was confirmed after reprogramming by sequencing DNA from all patient iPS cell lines (Supplementary Figure S2B).

### Differentiation of iPS Cell Lines Into Forebrain Neurons

Induced pluripotent stem cells were differentiated to forebrain neurons using a dual SMAD inhibition protocol (Figure 1H), previously described (Chambers et al., 2009). By day 40 of differentiation (Figure 1I), patient and control iPS cell lines generated neurons that were positive for β-tubulin type III, a neuronal marker; FOX G1, a forebrain neuron marker; and SYN1, a neuronal synaptic marker (Figure 1I). There was no difference in the differentiation ability between patient and control cell lines (Figure 1I).

### Spastin Was Reduced in Patient Neurons

**FIGURE 2:**
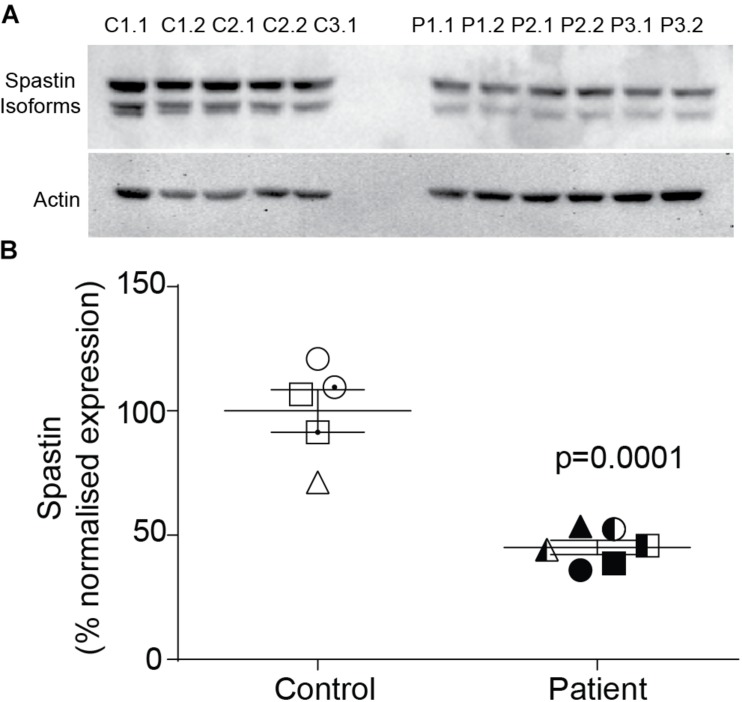
Spastin expression is reduced in neurons of *SPAST* patients. **(A)** Western blot analysis of spastin expression in patient and control neurons showing both isoforms. **(B)** Expression of spastin isoforms relative to actin.

Compared to control neurons, patient neurons had reduced expression of spastin (Figure 2A). Western blot analysis showed that spastin expression in patient neurons was about 50% of control neurons (Figure 2B; control cells: 100 ± 8.59%, patient cells: 45.1 ± 2.89%, *t* = 6.54, df = 9, *p* = 0.0001).

### Reduced Acetylated α-Tubulin Expression and Increased Axon Swellings in Patient Axons

**FIGURE 3:**
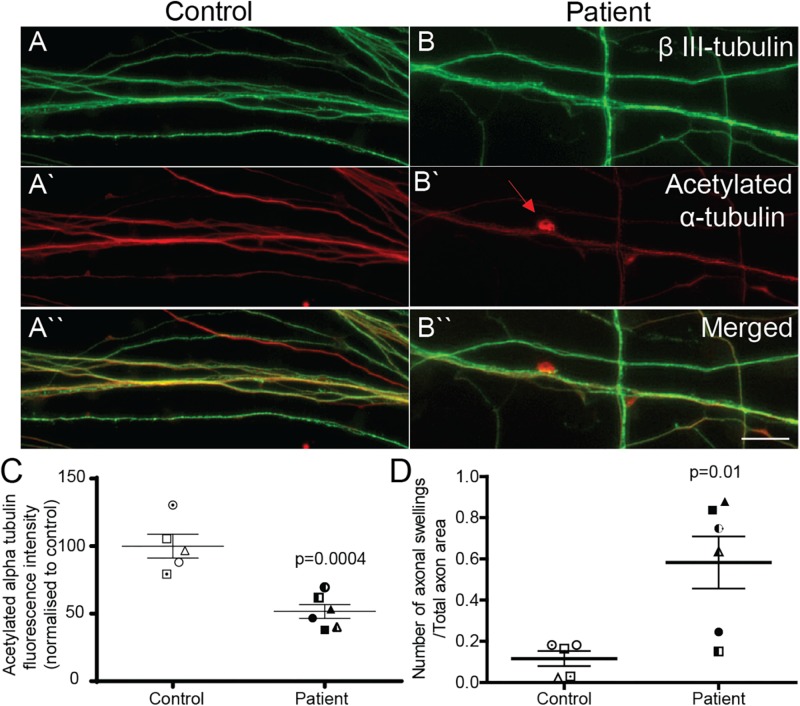
Acetylated α-tubulin expression is reduced and axon swellings are increased in *SPAST* patient neurons. Control **(A,A‘,A“)** and patient **(B,B‘,B“)** axons co-immunostained for β III-tubulin, neuronal marker, and acetylated α-tubulin. Scale bar = 100 μm. Arrows, axon swelling. **(C)** Quantification of fluorescence intensity of acetylated tubulin in control and patient axons. **(D)** Quantification of axon swelling density in control and patient axons.

Patient and control axons were identified as long processes immunostained for the neuron marker, β III-tubulin (Figures 3A,B). Levels of stabilized microtubules were quantified by measuring the fluorescence intensity of acetylated α-tubulin protein (Figures 3A‘,B‘). Compared to control axons, patient axons had reduced expression of acetylated α-tubulin (Figure 3C; control cells: 100 ± 8.76%, patient cells: 51.63 ± 5.06%, *t* = 4.991, df = 9, *p* = 0.0004).

Axon swellings in control axons were rarely observed (Figure 3A‘), but were often observed in patient axons (Figure 3B‘). Axon swelling density is the ratio of the number of axonal swellings to total axon area. The density of axon swellings was significantly greater in patient axons than in controls (Figure 3D; control axon swelling density: 0.1163 ± 0.037, patient axon swelling density: 0.5828 ± 0.1267, *t* = 3.240, df = 9, *p* = 0.01).

### Reduced Number of Peroxisomes in Patient Axons

To test if patient axons have reduced number of organelles whose transport is dependent on microtubules, we quantified the number of peroxisomes in control and patient axons. Compared to control axons, patient axons had 28% fewer peroxisomes (Supplementary Figure S3; peroxisomes per control axon: 79.60 ± 8.524, peroxisomes per patient axon: 57.11 ± 6.907, *t* = 2.074, df = 9, *p* < 0.05).

### Reduced Microtubule-Dependent Peroxisome Axon Transport

**FIGURE 4:**
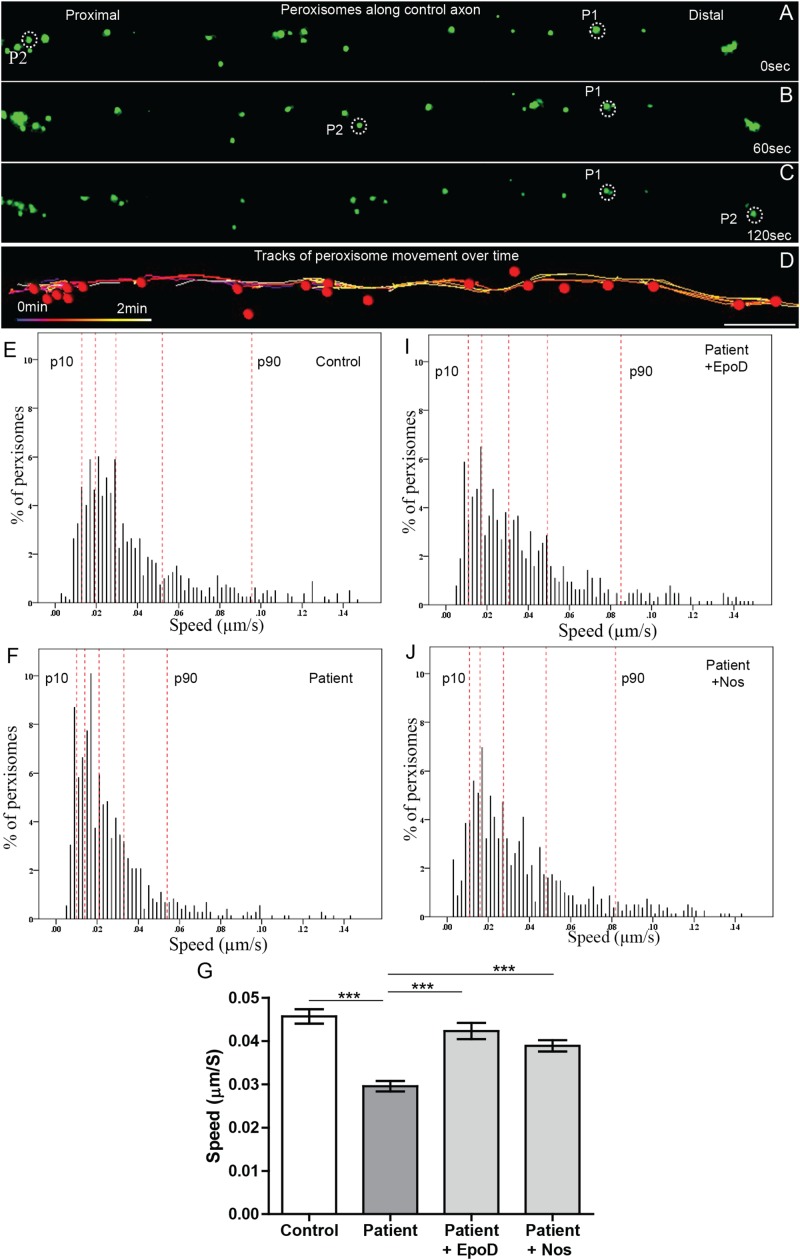
Peroxisome transport is reduced in *SPAST* patient axons and rescued by tubulin-binding drugs. **(A,B,C)** Time-lapse images of peroxisomes (green) in control axon at 0, 60, and 120 s (times shown on right). **(D)** Peroxisomes in **A** (red dots) and their paths of movement (colored lines) identified by the automated analysis software. Scale bar = 10 μm for all images. Frequency distributions of peroxisome speeds of: **(E)** untreated control group, **(F)** untreated patient group, **(I)** epothilone D-treated patient group, and **(J)** noscapine-treated patient group. The red dotted lines indicate the 10th, 25th, 50th, 75th, and 90th percentiles of the frequency distributions. **(G)** Means of peroxisome speed distributions. ***Different from the untreated patient group *p* < 0.0001.

Peroxisome transport is essential to maintain axon integrity (Bottelbergs et al., 2010) as they regulate oxidative state via detoxification of hydrogen peroxide by catalase (Antonenkov et al., 2010). To test if peroxisome transport along patient axons is impaired, we performed live cell imaging and tracked their movement. Peroxisome movement along the axons was quantified using time-lapse imaging of GFP-labeled peroxisomes (Figures 4A–C). Peroxisomes exhibit local Brownian movements (indicated by peroxisome P1) and saltatory movements along the axon (indicated by peroxisome P2). The time-lapse movies were analyzed using automated image analysis software, IMARIS. Figure 4D shows peroxisomes identified as “spots” (red dots) and their paths of movement as identified by the analysis software. The speeds of peroxisome transport were calculated for about 1500 peroxisomes from six patient- and five control iPS cell lines differentiated neurons (∼130 peroxisomes per cell line).

Peroxisomes exhibited typical Brownian and saltatory movements as described in olfactory neural stem cells and other mammalian cells (Figures 4A–C and Supplementary Videos S1, S2) (Huybrechts et al., 2009; Wali et al., 2016). The frequency distribution of peroxisome speeds from patient and control axons had similar positively skewed non-normal distributions (skewness > 1) (Figures 4E,F), indicating that most peroxisomes traveled at slow speeds with few peroxisomes traveling at fast speeds. Fast peroxisome transport (with speeds above 90th percentile) is microtubule dependent (Wiemer et al., 1997; Wali et al., 2016). The average speed of peroxisome transport in patient axons was 35% less than in control axons (control peroxisome speed: 0.046 ± 0.001 μm/s; patient peroxisome speed: 0.028 ± 0.001 μm/s). Most of this difference appeared to be due to the reduced number of fast moving peroxisomes with concomitant increase in slow moving peroxisomes in patient cells, seen in the shift to the left of the frequency distribution of peroxisome speeds and the changes in the speeds represented by the percentiles (Figures 4E,F). Quantile regression analysis of the frequency distributions showed a significant reduction (*p* < 0.001) at all percentiles in mean peroxisome speeds in the patient group compared to control group (Table 1A) based on analysis of speeds of 715 peroxisomes from five control cell lines and 671 peroxisomes from six untreated patient cell lines. The biggest difference is seen in the fastest peroxisomes where the 90th percentile of the patient peroxisome speeds is approximately equivalent to the 75th percentile of the control peroxisome speeds (Figures 4E,F).

**TABLE 1:** Quantile regression of frequency distributions of peroxisome speeds.

**Percentiles**	**β**	**SE**	***P*-value**	**95% CI**	
**(A) Control vs patient peroxisomes**					
q10	–0.0035	0.0005828	<0.001	–0.0046433	–0.0023567
q25	–0.0055	0.0008333	<0.001	–0.0071346	–0.0038654
q50	–0.0075	0.0012936	<0.001	–0.0100376	–0.0049624
q75	–0.0145	0.0020909	<0.001	–0.0186017	–0.0103983
q90	–0.0355	0.0046723	<0.001	–0.0446655	–0.0263345
**(B) Control vs epothilone D treated patient peroxisomes**					
q10	–0.001	0.0003164	0.002	–0.0016207	–0.003793
q25	–0.001	0.000405	0.0014	–0.0017945	–0.0002055
q50	0.00075	0.0007972	0.347	–0.000814	0.002314
q75	0.00075	0.0012249	0.54	–0.0016531	0.0031531
q90	–0.003	0.0038045	0.431	–0.0104637	0.0044637
**(C) Control vs noscapine treated patient peroxisomes**					
q10	–0.00083	0.002299	<0.001	–0.0003824	–0.0003824
q25	–0.00117	0.0002562	<0.001	–0.0016693	–0.000664
q50	–0.00067	0.0004372	0.127	–0.0015242	0.0001909
q75	–0.0005	0.0009009	0.579	–0.0022673	0.0012673
q90	–0.00367	0.0020066	0.068	–0.0076029	0.0002695

*Quantile regression analysis comparing the frequency distributions of untreated control group compared to untreated patient group **(A)**, epothilone treated patient group **(B)**, and noscapine treated patient group **(C)**. For inter-percentile comparisons, the group difference (β), standard error (SE), significance (*p*-value), and 95% confidence interval (CI) values are shown. (*N* = 715 peroxisomes from five control cell lines, *N* = 671 peroxisomes from six untreated patient cell lines, *N* = 562 peroxisomes from six epothilone-treated, and *N* = 716 noscapine-treated patient cell lines). Treatment of patient group restored the peroxisome speeds of the faster peroxisomes, seen as no difference in speeds above the 50th percentile.*

### Tubulin-Binding Drugs Rescued Peroxisome Transport in Patient Axons

To test if tubulin-binding drugs can rescue the reduced peroxisome axon transport in patient axons, patient axons were treated with tubulin-binding drugs epothilone D and noscapine and their peroxisome transport was compared to peroxisome transport of control axons. Both drug treatments significantly increased the average peroxisome speeds in patient axons back to control axon levels (*p* < 0.001) (Figures 4E–G,I,J). Drug treatments shifted frequency distributions of peroxisome speeds to the right, increasing the numbers of fast-moving peroxisomes after drug treatment to values similar to controls (Figure 4E). Quantile regression analysis of the frequency distributions showed a rightward shift in the 50th percentile and above, indicated by the lack of difference in these percentiles after drug treatment compared the controls (Tables 1B,C), based on analysis of speeds of 562 peroxisomes from six epothilone-treated and 716 peroxisomes from six noscapine-treated patient cell lines.

### Patient Axons Were More Susceptible to Fragmentation Induced by Hydrogen Peroxide

**FIGURE 5:**
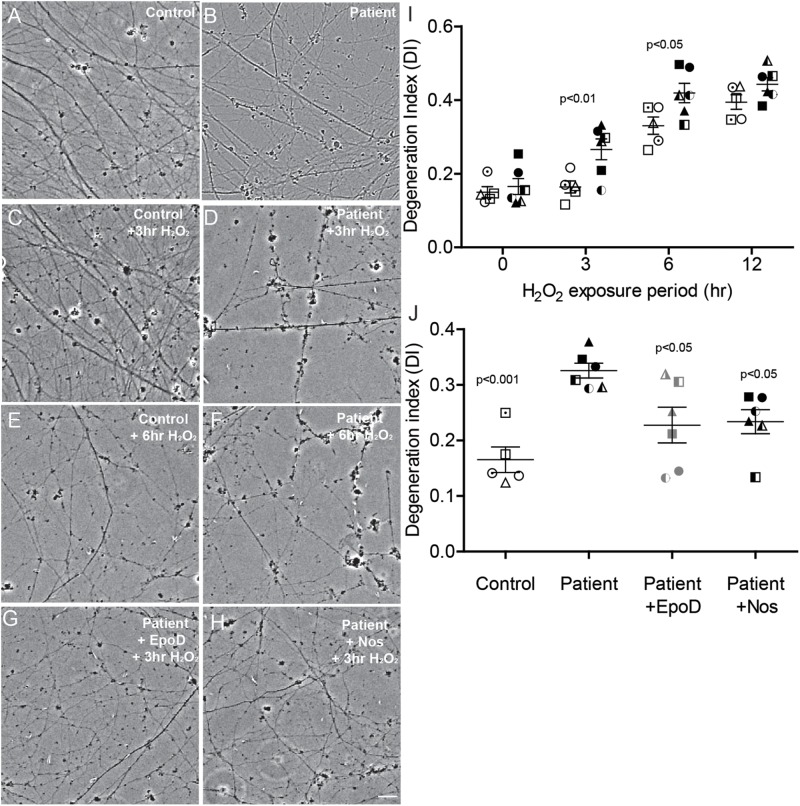
Axon degeneration under oxidative stress increased in *SPAST* patients and is rescued by tubulin-binding drugs. **(A,B)** Untreated control and patient axons. Control and patient axons exposed to 20 μM H_2_O_2_ for 3 h **(C,D)** and 6 h **(E,F)**. Patient axons treated with epothilone D **(G)** or noscapine **(H)** for 24 h before exposing to 20 μM H_2_O_2_ for 3 h. Scale bar = 25 μm. I: Degeneration index (DI) at different periods of treatment H_2_O_2_ exposure. Patient DIs (filled symbols) were significantly different from control DIs (open symbols) at 3 and 6 h. **(J)** DIs of untreated controls (open symbols) and untreated patients (filled symbols) and patient cultures treated with epothilone D or noscapine for 24 h prior to 3 h exposure to 20 μM H_2_O_2_. Statistics shown for differences between untreated patient axons and other groups.

It has been previously established that damage to the microtubule network of cells, particularly neurons, sensitizes the cell to further damage by oxidation (Fukui, 2016). To examine whether the microtubule defects observed here in *SPAST* patient neurons recapitulates this effect, we exposed the neurons to oxidative stress induced with hydrogen peroxide. Axon fragmentation was subsequently quantitated using an image-based assay, as previously described (Sasaki et al., 2009). Healthy axons are continuous, whereas degenerating axons are fragmented or beaded, and in this assay axon fragmentation is quantified and reported as the DI, which ranges from 0 (intact axons) to 1 (completely beaded/fragmented axons) (Sasaki et al., 2009). At baseline, the untreated control and untreated patient axons were intact (Figures 5A,B). After exposure to hydrogen peroxide, patient and control axons fragmented with fragmentation increasing with time (Figures 5C–H,I). A repeated measures analysis of variance showed a significant main effect of disease status (*p* = 0.0003) and a significant main effect for hydrogen peroxide treatment (*p* < 0.0001). Dunnett’s *post hoc* multiple comparison indicated a significant increase in axon degeneration by patient neurons over controls after exposure to hydrogen peroxide for 3 h (*p* < 0.01) and 6 h (*p* < 0.05).

### Tubulin-Binding Drugs Reduced Hydrogen Peroxide-Induced Axon Fragmentation in Patient Axons

Patient cultures treated with epothilone D or noscapine for 24 h before exposure to hydrogen peroxide for 3 h had axon fragmentation similar to untreated controls (Figures 5A,G,H). DI was significantly greater in untreated patient axons than in untreated control axons (DI: 0.326 ± 0.013 vs 0.165 ± 0.023, respectively; Figure 5J). Treatment with epothilone D and noscapine decreased DI in patient axons toward untreated control axon levels (Figure 5J). ANOVA indicated a significant effect of treatment (*p* < 0.01). Dunnett’s *post hoc* multiple comparison tests indicate that untreated patient axon degeneration was significantly greater than control axon degeneration (*p* < 0.001). DI of patient axons was significantly reduced by treatment with epothilone D (*p* < 0.05) and noscapine (*p* < 0.05).

## Discussion

We show here that iPS cell-induced neurons from a variety of *SPAST* HSP patients carrying different mutations, including frameshift, missense, and exonic deletion mutations, share common axon pathologies of reduced stabilized microtubules, increased density of axon swellings, reduced microtubule-dependent peroxisome transport, and reduced number of peroxisomes. Patient axons did not show signs of degeneration, evaluated by quantifying axon fragmentation under basal conditions. However, patient axons fragmented significantly more than controls following hydrogen peroxide exposure, suggesting for the first time that the *SPAST* patient axons are more sensitive than controls to the deleterious effects of oxidative stress.

Our finding of reduced axonal transport in iPS-neurons derived from *SPAST* patients concurs with findings of studies involving post-mortem spinal cord analysis (Kasher et al., 2009) and iPS-neurons (Denton et al., 2014; Havlicek et al., 2014), showing that we were able to recapitulate HSP pathology in our neurons. Patient axons have less spastin, encoded by *SPAST*. This effect is commonly seen in patient-derived iPS-neurons (Denton et al., 2014; Havlicek et al., 2014) and ONS cells (Abrahamsen et al., 2013) and presumed to be a haploinsufficiency effect (Abrahamsen et al., 2013). Since spastin is involved in maintaining microtubule dynamics, reduced spastin is likely to affect cellular processes dependent on microtubules including organelle transport. Consistent with this, patient axons have reduced mean speed of peroxisome transport compared to controls. The reduced mean speed of peroxisome transport was a consequence of reduced number of fast microtubule-dependent peroxisome movements, as treatment of patient axons with tubulin-binding drugs, increased the number of fast microtubule-dependent peroxisome movements and subsequently increased the mean peroxisome transport speeds in patient axons to levels comparable with control axons, thus rescuing the axonal transport deficit. Also using patient ONS cells, we have previously shown that the same concentration of tubulin-binding drugs epothiloneD (2 nM) and noscapine (10 μM) used here increase mean speed of peroxisome transport by increasing the levels of stabilized microtubules (Fan et al., 2014). It is possible that both the tubulin-binding drugs may have independent mechanisms by which they increase peroxisome transport speed and increasing levels of stabilized microtubules is one possibility. For example, organelle transport speed can be increased by reintegration of microtubule lattice with drug treatment in addition to increased stabilized microtubules (Kan et al., 2011).

It has been previously established that damage to the microtubule network of cells, particularly neurons, sensitizes the cell to further damage by oxidation (Fukui, 2016). Is this the case with *SPAST* patient neurons? Oxidative stress arises from a significant increase in concentrations of reactive oxygen species and/or a decrease in the detoxification mechanisms. Coincidently, both cellular organelles, peroxisomes and mitochondria, whose transport is shown to be reduced in *SPAST* HSP, here and elsewhere (Abrahamsen et al., 2013; Denton et al., 2014; Havlicek et al., 2014), are involved in maintaining reactive oxygen species levels in cells. It is likely that slow or reduced transport of peroxisomes/mitochondria could limit the availability of these organelles, particularly at the axon distal ends, making them unavailable to manage increased reactive oxygen species when necessary. Consistent with this, exposure to hydrogen peroxide induced axon fragmentation in both patient and control axons; however, patient axons displayed significantly increased fragmentation. There was no difference between the patient and control axons at baseline, showing that oxidative stress can have deleterious effects on *SPAST* patient axons. Treatment of patient axons with the same concentrations of epothilone D and noscapine drugs that rescued the axonal transport deficit also protected the patient axons from oxidative stress-induced increased fragmentation showing for the first time that *SPAST* patient axons are vulnerable to oxidative stress-induced degeneration as a consequence of reduced axonal transport.

We show here that reduced axon transport and oxidative stress-induced axonal fragmentation are common axon pathologies in iPS-neurons derived from a variety of *SPAST* patients carrying different mutations. This understanding is relevant when testing drug candidates to rescue axon pathologies. A common drug treatment may be effective across HSP patients carrying different *SPAST* mutations, as seen here with both epothilone D and noscapine. We believe this is an extremely promising strategy to find a candidate drug that can rescue *SPAST* related axon pathologies *in vitro*.

## Data Availability Statement

All datasets generated for this study are included in the article/Supplementary Material.

## Ethics Statement

Our study involving human participants was reviewed and approved by the Human Research Ethics Committee affiliated to the Northern Sydney Local Health District, New South Wales government, Australia. The ethics committee reference number: RESP/15/314. The patients/participants provided their written informed consent to participate in this study.

## Author Contributions

GW, AM-S, and CS conceived and designed the experiments and wrote the main manuscript text. GW generated and charecterized all the iPS cell lines, differentiated iPS cells to forebrain neurons, and performed all assays described in the manuscript. EL provided assistance with the western blot assay. NB provided advice and assistance with iPS generation and charecterization. J-SP performed the DNA sequencing experiments. RS assisted with live cell peroxisome transport assay data. GW, AM-S, and RS performed the statistical analysis. AM-S, CS, and GW provided funding for the research. All authors contributed to and edited the manuscript.

## Conflict of Interest

The authors declare that the research was conducted in the absence of any commercial or financial relationships that could be construed as a potential conflict of interest.
