# Supplementary material for: Oxidative Stress-Induced Axon Fragmentation Is a Consequence of Reduced Axonal Transport in Hereditary Spastic Paraplegia SPAST Patient Neurons
Source: Front Neurosci. 2020 May 7;14:401. doi: 10.3389/fnins.2020.00401 (PMC7221066; doi:10.3389/fnins.2020.00401)
Supplement: Supplementary file 1 [file Data_Sheet_1.docx]

**Supplementary table, figure legends and methods**

# Supplementary Table S1: List of patient and control participants

Genetic analysis of patients previously reported ^3^.

| **Patient ID** | **Exon** | **Nucleotide change** | **Mutation type** | **iPS cell lines** |
| --- | --- | --- | --- | --- |
| HSP patient with *SPAST* mutations | | | | |
| 09061 | 9 | c[1214_1215insT] | Frameshift mutation, Exon 9 skipped | Patient 1.1, patient 1.2 |
| 09062 | 12 | c.1466C>G | Missense | Patient 2.1, patient 2.2 |
| 09065 | 8–9 | - | Exonic deletion | Patient 3.1, patient 3.2 |
| Healthy controls | | | | |
| 10027 |  |  |  | Control 1.1, control 1.2 |
| 10022 |  |  |  | Control 2.1, control 2.2 |
| 10014 |  |  |  | Control 3.1 |

**Supplementary figure legends**

**Supplementary Figure S1: Pluripotency and differentiation capacity of patient and control induced pluripotent stem (iPS) cell lines**

TaqMan® hPSC Scorecard (Thermofisher, A15872) assesses pluripotency and tri-lineage differentiation potential using a qPCR gene expression panel analysed against a standardised reference set. Patient and control iPS cells (as indicated) were evaluated using this Scorecard assay in their undifferentiated iPS state as well as after embryoid body differentiation (EB). Control, Control3, Control5 refer to Controls 1.1, 2.1, 3.1 and Patient1, Patient3, Patient 5 refer to Patients 1.1, 2.1, 3.1, respectively. Mean gene expression fold change values for each of the cell lines in pluripotent state and in embryoid bodies is presented. Red: Upregulated; Blue: Downregulated. Embryoid body differentiation downregulated self-renewal genes (blue) and upregulated differentiation marker genes (red), especially ectodermal genes demonstrating capacity for neuronal differentiation of all cell lines.

**Supplementary Figure S2: Genetic analysis of iPS cell lines**

**A:** All six patient and five control iPS cell lines had normal karyotypes. **B:** Specific *SPAST* gene mutations present in patient-derived iPS cell lines.

**Supplementary Figure S3: Number of peroxisomes in patient and control axons**

Compared to control axons, patient axons had ~28% fewer peroxisomes per axon.

**Supplementary video 1**

Time-lapse movie of peroxisome movement along control axons for total duration of two minutes.

**Supplementary video 2**

Time-lapse movie of peroxisome movement along patient axons for total duration of two minutes.

**Supplementary methods**

**Generation of induced pluripotent stem cell lines**

# *Skin fibroblast biopsy and culture*

Skin fibroblasts were taken from the medial aspect of the forearm, and were cultured in Dulbecco’s Modified Eagle Medium (DMEM)/F12 (Gibco) with 10% fetal bovine serum at 37°C and 5% CO_2_. All cell lines were tested and were found to be negative for mycoplasma.

# *Reprogramming skin fibroblasts to iPS cell lines*

Human iPS cells were established from skin fibroblast cells using the CytoTune®-iPS 2.0 Sendai Reprogramming Kit (A16518, Life Technologies) as per the manufacturer’s manual. Briefly, human fibroblasts at low passage number (5 or lower) were cultured in Dulbecco’s modified Eagle’s medium (DMEM) supplemented with 10% foetal bovine serum (FBS) ESC-Qualified (16141-079, Life Technologies), MEM Non-Essential Amino Acids Solution, 10 mM (11140-050, Life Technologies) and β-mercaptoethanol, 55 mM (21985-023, Life Technologies) in a 6-well plate until they were ~80% confluent. On day 0, the cells were transduced with reprogramming vectors *OCT 3/4*, *SOX 2*, *KLF 4* and *c-MYC* provided in the CytoTune-iPS Sendai reprogramming kit. On day 1, fibroblast medium was replaced to remove the reprogramming vectors. Between days 2-6, the fibroblast medium was replaced every other day. On day 7, the transduced cells were plated on irradiated mouse embryonic fibroblasts (MEFS, S1520-100, Gibco). On day 8, the fibroblast medium is switched to iPS cell medium i.e. DMEM/F-12, GlutaMAX™ supplement (10565018, Life Technologies), KnockOut™ Serum Replacement (10828-028, Life Technologies), MEM Non-Essential Amino Acids Solution 10 mM (11140-050, Life Technologies), β-mercaptoethanol (55 mM, 21985-023, Life Technologies) and FGF2 (10 ng/mL, PHG0264, Life Technologies). Between days 9-28, iPS cell medium was replaced every other day. iPS cell colonies appeared around day 15. After 28 days, iPS cell colonies positive for a pluripotent marker SSEA4 (Fig. 1B) were manually removed under a stereo microscope and transferred onto fresh MEF culture dish for expansion under feeder-dependent conditions.

# *Selecting SSEA-4 positive iPS colonies to establish iPS cell lines*

The human iPS selection kit (SCR502, Millipore) was used for live cell staining and identification of reprogrammed iPS cells from a heterogeneous population of reprogrammed iPS cells (fully and partially reprogrammed iPS cells). The staining was performed as per manufacturer’s instructions. iPS cells were cultured in iPS cell medium in 6 well plates. The conjugated antibody was diluted appropriately in iPS cell medium (mouse anti-SSEA-4: 1/250 dilution based on 0.25 mg/mL) and iPS cells were incubated with the antibody for 30 minutes in 37ºC and 5%CO_2_. Then, the cells were washed 3 times with 2 mL fresh iPS cell medium. iPS cells were viewed under a fluorescence microscope and positively labelled iPS cells were passaged to a new 6 well plate containing MEFs (irradiated) and iPS cell medium. The cells were then cultured, expanded and assessed for pluripotency and differentiation ability.

***Flow cytometry analysis of pluripotency markers SSEA-4 and Sox2***

iPS cell lines generated were assessed for pluripotency markers using flow cytometry. Briefly, iPS cell colonies were disaggregated using Gentle Cell Dissociation Reagent (Cat. #07174, Stemcell technologies) and plated on gelatine for 1hour at 37°C to remove mouse embryonic feeder cells. The non-adherent iPS cells were then passed through a 0.70 µm filter to get consistent single cell suspension. Cells were labelled with antibodies from Miltenyibiotec according to manufacturer’s instructions: SSEA4 (Cat #: 130-098-371), Sox2 (130-104-993), isotype controls REA Control (S) antibody (130-113-437) and REA Control (I) antibody (130-118-354). Cells were permeabilised and fixed using the BD Cytofix/Cytoperm™ soultion for Sox 2 intracellular staining. Fluorescence was quantified on the BD LSR Fortessa flow cytometer and quantified with BD FACSDiva™ software.

***Immunostaining of pluripotency marker Sox2***

iPS cells were fixed in 4% paraformaldehyde for 10 minutes at room temperature. This was followed by permeabilization with 0.1% triton-X in HBSS containing 3% bovine serum albumin (Sigma) for 30 minutes, and incubated with an antibody against Sox2 (ab97959, Abcam). The cells were then washed in HBSS and incubated with an appropriate 488 Alexa-dye conjugate (Thermofisher Scientific) secondary antibody for 30 minutes.

***TaqMan® hPSC Scorecard™ Panel to evaluate gene expression of iPS cell lines***

TaqMan® hPSC Scorecard™ Panel (A15872, Life Technologies) was used to evaluate the gene expression of human iPS cells as per manufacturer’s instruction. Briefly, RNA was extracted from undifferentiated iPS cells and after differentiated into EBs using the RNeasy Mini Kit (74104, Qiagen) as per manufacturer’s instructions. Isolated RNA was used to generate cDNA in a reverse transcription reaction using the High-capacity cDNA Reverse Transcription Kit with RNase Inhibitor (4374966, Life Technologies) and TaqMan® hPSC Scorecard™ Panel was used to quantitate RNA expression levels of genetic markers for pluripotency and differential potential. Gene expression data was analysed using the Scorecard™ Analysis Software, available at [www.lifetechnologies.com/scorecarddata](http://www.lifetechnologies.com/scorecarddata). The assay was performed on one clone from each iPS cell line generated in their pluripotent iPS cell state and after random differentiation to embryoid bodies. The cell lines tested: Control 1.1, Control 2.1, Control 3.1, Patient 1.1, Patient 2.1 and Patient 3.1.

**Sanger sequencing to confirm patient mutation** Mutations in *SPAST* HSP patient-derived iPS cells were confirmed by direct. Briefly, small fragment of genomic DNA isolated from iPS cells was amplified in a PCR using a specific primer set for each mutation: c.1214_1215insT; 5’-TTTTCCTATTAAATGGCCAAGG-3’ and 5’-CATGAAGCCCAATAAAAGACG-3’, c.1466C>G; 5’-CCTGGCCTCATAGCTTACATTT-3’ and 5’-CCTGCCAACATTTTGACTGAT-3’ and deletion of exon 8-9; 5’-GGAATGTGGACAGCAACCTT-3’ and 5’-TCTTGTGGCCTATTAGTTGCAC-3’. Each target PCR product was purified and employed in a sequencing reaction using a Bigdye terminator v3.1 kit (Applied biosystems) and an aforementioned primer, and the sequence was acquired by capillary separation on an AB 3730xl (Applied biosystems) at Australian Genome Research Facility.

***Cytology to evaluate chromosomal integrity of iPS cell lines*** The process of cellular reprogramming and the maintenance of pluripotent cells in culture can lead to chromosomal defects. To exclude this, cytogenetic analysis was performed on G-banded metaphase cells (Cytogenetics Department, Royal North Shore Hospital, Sydney).

**Differentiation of induced pluripotent stem cell lines *Spontaneous differentiation of iPS cells to embryoid bodies*** Embryoid bodies were generated in accordance to previously published protocols ^51^. Briefly, iPS cells were cultured and grown in 6 well plates on MEFs to ~90% confluence. iPS cell colonies were divided into clumps of ~ 300 cells and resuspended in iPS cell medium but without FGF2, in a 6 well ultra-low attachment plate (CLS3471, Sigma). Fresh medium was replaced every 3-4 days for 15 days. The low-binding plates promoted the iPS cells to form aggregates in suspension. ***Neuronal induction and differentiation*** Patient and control iPS cells were differentiated into FOXG1+ forebrain neurons following a previously published protocol^18,19^. Briefly, iPS cell cultures were disaggregated using Accutase (A1110501, Life Technologies) and pre-plated on gelatine for 1hour at 37°C in the presence of Y-27632 RHO/ROCK pathway inhibitor (72302, StemCell Technologies) to remove mouse embryonic feeder cells. The non-adherent cells were then collected and plated on Matrigel coated dishes in MEF-conditioned medium along with 10ng/ml FGF2 and ROCK-inhibitor. ROCK inhibitor was withdrawn and cells were cultured in cell medium for 2-3 days or until they were confluent. For days 0 to 5, cells were cultured in knockout serum replacement media with 10 µM TGFβ inhibitor (Tocris) and 100nM ALK2/ALK3 inhibitor (Stemgent). After 5 days of differentiation, the TGFβ inhibitor was withdrawn and increasing amounts (25%, 50%, 75%) of NBS1 media (Neurobasal medium, Thermofisher scientific; 100x L-Glutamine, Thermofisher scientific and 50x Neurocult SM1, Stem cell technologies) was mixed with knockout serum replacement medium every day, maintaining the 100nM ALK2/ALK3 inhibitor. Cells were replated (passage 1) in NBS1 media onto poly-D-lysine (Sigma) coated culture dishes in the presence of brain-derived neurotrophic factor, ascorbic acid, glial cell-derived neurotrophic factor, DAPT and dibutyryl cAMP. Second passage (passage 2) was performed between day 25 and 29, using Accutase to dissociate into single cells. Following second passage, cells were continued in NBS1 medium with brain-derived neurotrophic factor, ascorbic acid, glial cell-derived neurotrophic factor and DAPT until ~ day 40.

***β III-tubulin, GATA4, Brachury, FOXG1, SYN1, Acetylated α-tubulin immunostaining***

Cells were fixed in 4% paraformaldehyde for 10 minutes at room temperature. This was followed by permeabilization with 0.1% triton-X in HBSS containing 3% bovine serum albumin (Sigma) for 30 minutes, and incubated with an antibody against β III-tubulin (T8660, Sigma) or Brachyury (ab20680, Abcam) or GATA4 (ab84593, Abcam) or SYN1 (ab8, Abcam) or FOXG1 (ab18259, Abcam) or Acetylated α-tubulin (ab24610, Abcam) for 2 hours at room temperature. Secondary antibodies were species-specific Alexa-dye conjugates (Thermofisher Scientific) for 30 minutes. For nucleus labelling, cells were stained with Hoechst (H3570, Life Technologies) for 10 minutes at room temperature.

**Phenotype of differentiated forebrain neurons**

# *Spastin expression in differentiated neurons*

Thirty µg of proteins were resolved by sodium dodecyl sulfate polyacrylamide gel electrophoresis on 12% polyacrylamide gels and electro-transferred onto nitrocellulose membranes, which were blocked in 5% non-fat milk powder in phosphate buffered saline with Tween 20 (PBST) for 1 hour at RT and incubated overnight at 4°C with the following primary antibodies diluted in 5% milk: Spastin Rabbit antibody (dilution 1/500; ABN368, Millipore), Actin mouse antibody (dilution 1/2000, #A5441, Sigma). Incubation with anti-mouse and anti-rabbit secondary antibodies coupled to horseradish peroxidase (dilution 1:2000 for Spastin, 1:4000 for Actin was performed at room temperature for 1 hour, followed by repeated washing with PBST. Immunoreactive bands were visualized using SuperSignal Femto Chemiluminescent Substrate (#34096; Pierce, Rockford, IL) according to the manufacturer's instructions on an ImageQuant RT ECL imaging system (GE Healthcare). Band intensities were measured using Image Lab software (Bio-Rad). Spastin intensity was normalized against Actin expression to obtain relative expression levels.

# *Axon swellings*

Axon swelling density was calculated as the number of axonal swellings divided by the total axon area in a field of view. Axons were defined as long, β III-tubulin positive neurites that were co-stained with acetylated α-tubulin, a marker for stabilised microtubules along axons ^20^. Immuno-labelling was done as described above with antibodies to acetylated α-tubulin (ab24610, Abcam) and β III-tubulin (T8660, Sigma) for 1 hour at room temperature. The numbers of axon swellings were counted manually and divided by the total length of β III -tubulin positive neurites in each field of view, which was measured using ImageJ (Version 1.51t). At least three field of views were randomly selected for each Patient and Control cell line. ***Peroxisome imaging and analysis in living cells***

Peroxisome imaging and analysis in neurons was performed as described previously ^12^. Neurons were cultured in 4 well Nunc™ Lab-Tek™ II Chambered cover glass (155382, Thermofisher scientific). To visualise peroxisomes in neurons, the neurons were transduced for 48 hours with a live-cell GFP peroxisome probe (1:200; C10604, CellLight Peroxisome-GFP BacMam 2.0; Life Technologies). Time-lapse movies were captured using a Leica SP5 confocal microscope (Leica, Germany) with images captured every 2 seconds for a total duration of 2 minutes. The exposure period was constant throughout the experiment. Image analysis software (Imaris, Bitplane) was used to track the movement of peroxisomes along the axons. The peroxisome movement analysis was fully automated, with no intervention by the experimenter, removing observer bias. The movies were quantified using semi-automated image analysis software (Imaris, Bitplane) as described previously ^12^. The mean speed (µm/s) was calculated for all peroxisomes tracked. Peroxisomes that were not tracked for the entire observation period were deleted from the analysis. Mean speeds of peroxisomes were calculated for 715 peroxisomes from untreated control axons, 671 peroxisomes from untreated patient axons, 562 peroxisomes from epothilone D-treated patient axons and 716 peroxisomes from noscapine treated-patient axons. These peroxisomes were analysed from time-lapse movies imaged from three independent wells for each clone. Since the intention was to rescue the peroxisome transport deficit (peroxisome speed) in patient axons to levels comparable to control axons, only patient axons and not control axons were treated with the drugs. Axons for live cell imaging of peroxisomes were defined as the largest neurite extending from the cell soma with at least 120 µm of the proximal end unobstructed by other neurites. Peroxisomes along 100µm of the axons starting 20µm from the cell body were quantified using Imaris. For peroxisome distribution analysis, the first frame of every time-lapse movie was used.

**Effect of oxidative stress on axon degeneration**

To induce oxidative stress, we used 20µM H_2_O_2,_ that causes axonal defects but not cell death^53^. Patient and Control axons were treated with H_2_O_2_ for 3h, 6h and 12h. H_2_O_2_ culture media had no phenol red or other anti-oxidants that can nullify H_2_O_2_ effect in cell culture. Patient cells were treated with 2nM epothilone D or 10µM noscapine for 24hrs prior to H_2_O_2_ treatment after which axon degeneration was quantified by image analysis giving an Degeneration Index (DI) as described previously ^23^. For axon degeneration assays, neurons were cultured in Neurobasal® Medium, minus phenol red (12348017, Thermofisher scientific) and B-27® Supplement (50X), minus antioxidants (10889038, Thermofisher scientific), with hydrogen peroxide media being replaced every hour.
