# Supplementary figures and images for: Oxidative Stress-Induced Axon Fragmentation Is a Consequence of Reduced Axonal Transport in Hereditary Spastic Paraplegia SPAST Patient Neurons
Source: Front Neurosci. 2020 May 7;14:401. doi: 10.3389/fnins.2020.00401 (PMC7221066; doi:10.3389/fnins.2020.00401)

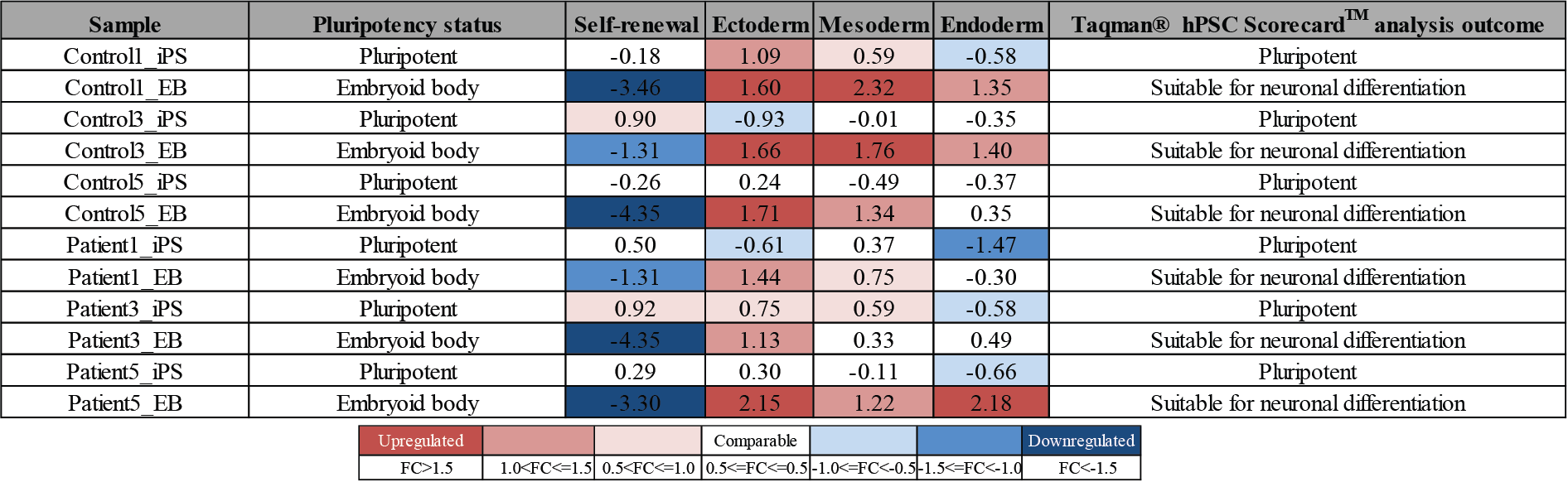

Supplement: Supplementary file 2 [file Image_1.TIF]

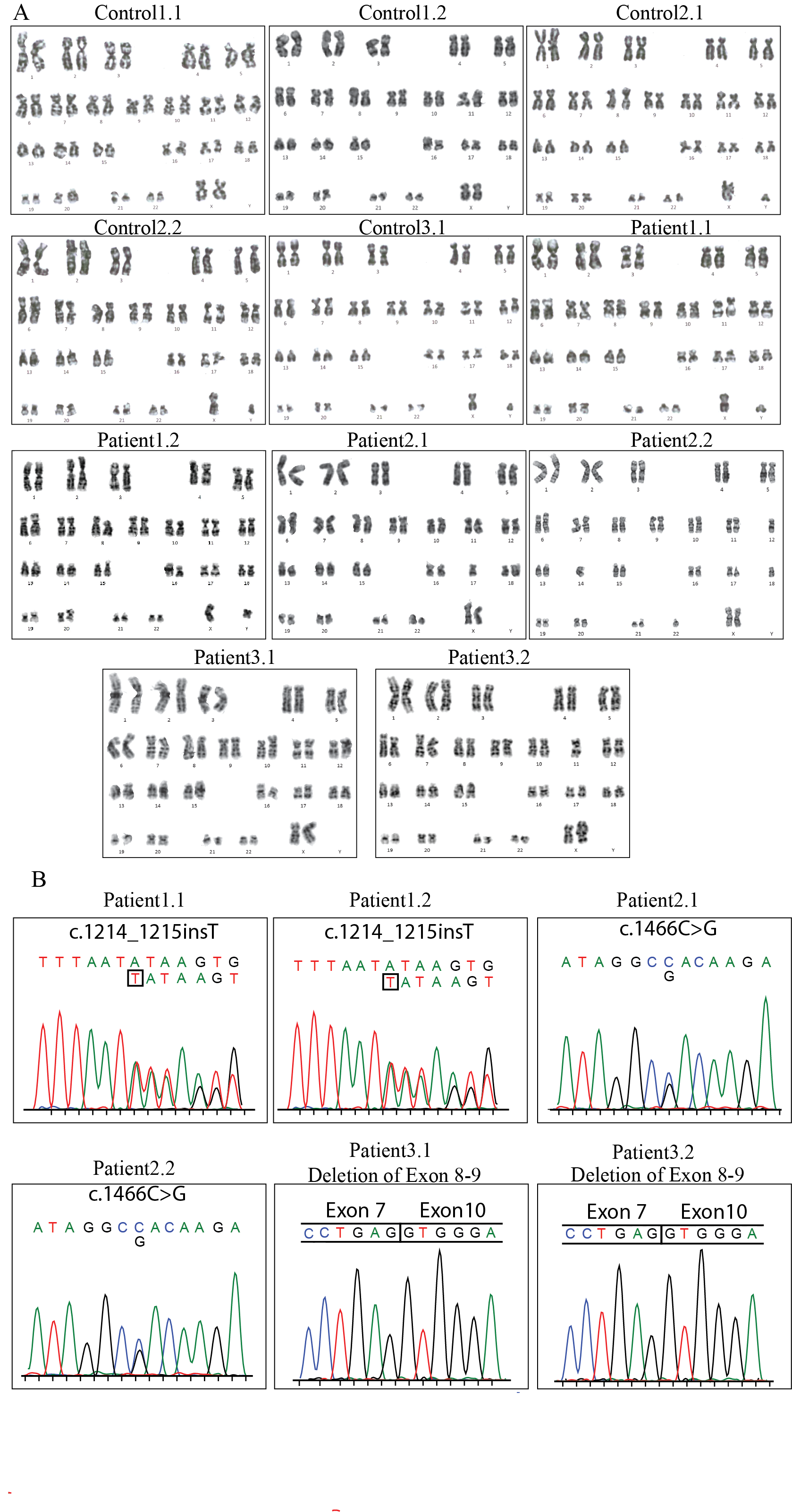

Supplement: Supplementary file 3 [file Image_2.TIF]

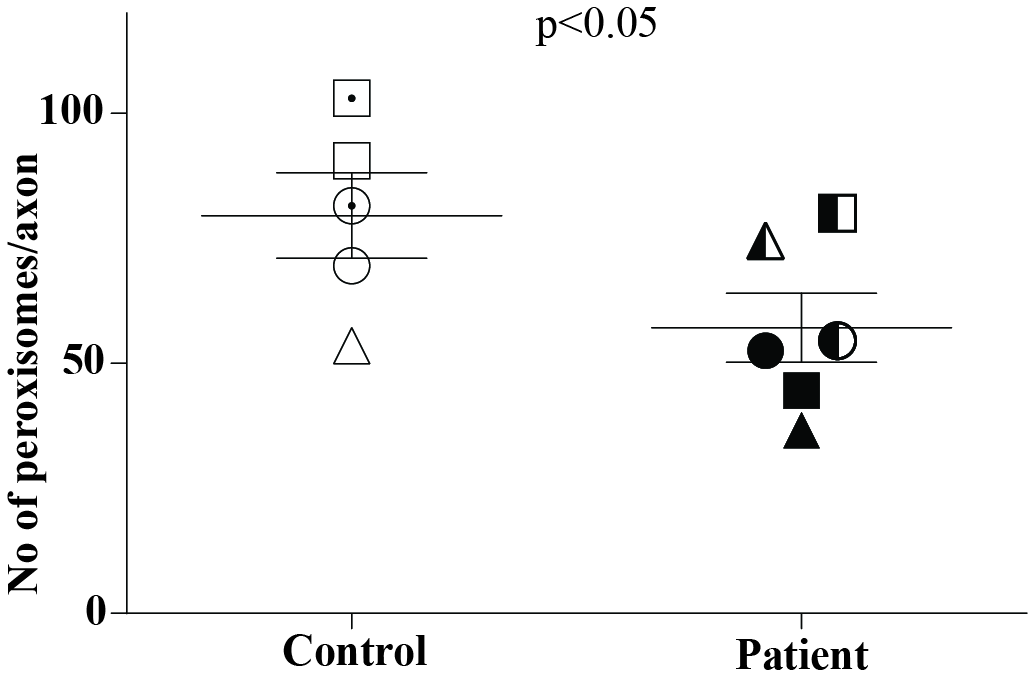

Supplement: Supplementary file 4 [file Image_3.TIF]
